# Effects of thermal stress, magnetic field and rotation on the dispersion of elastic waves in an inhomogeneous five-layered plate with alternating components

**DOI:** 10.1177/0036850420940469

**Published:** 2020-07-17

**Authors:** Rahmatullah Ibrahim Nuruddeen, Rab Nawaz, Qazi Muhammad Zaigham Zia

**Affiliations:** 1 Department of Mathematics, COMSATS University Islamabad, Islamabad, Pakistan; 2 Department of Mathematics, Faculty of Science, Federal University Dutse, Dutse, Nigeria

**Keywords:** Surface waves, magnetic field, rotation, thermal stress, layered plate

## Abstract

This study explores the effects of the thermal stress, rotation, and magnetic fields on the propagation of surface waves in a symmetric five-layered inhomogeneous elastic plate. The harmonic wave solution was adopted to reduce the given partial differential equations to their corresponding ordinary differential equations, which were later solved using a classical method. The Mathematica software was also employed for the numerical simulations. The dispersion relations associated with both the symmetric and antisymmetric modes have been determined and analyzed with regard to the aforementioned external effects and rotation. The variations in thermal stress positively affect the wave propagation in the plate even for relatively smaller values of the temperature variation. Also, the same trend is observed in the case of the magnetic field, but for higher values. Finally, for the rotation, it was found that the angular velocity must reach at least 10^4^ before a significant change is observed; which in fact differs from the situations of half-plane reflection and single-layered.

## Introduction

Propagation of elastic waves in various structures has been extensively examined in the past decades due to its frequent occurrence in nature and encounters in different phenomena of science and engineering applications. In particular, fields like the geology, modern aerospace, earthquakes, plasma physics and automotive industries to mention a few have long acknowledged the presence and dynamicity of wave propagation phenomena.^1–4^ Moreover, several studies in favour of the present topic have long been established with regard to the influence of certain external forces that significantly alter the propagation of waves in different bodies. For instance, the effect of magnetic field forces on the propagation of waves in elastic bodies was studied in Chadwick^5^ and Kaliski and Petykiewicz,^6^ while the thermal stress effects on the propagation of waves in an initially stressed anisotropic plate and double-layered nano-plates were presented by Selim^7^ and Wang et al.,^8^ respectively. Furthermore, the propagation and reflection of surface waves in the presence of rotation have been analysed in a layer resting on thermo-diffusive elastic half-plane in Kumar and Chawla^9^ and in an elastic stress free-surface half-space with thermal relaxation,^10,11^ respectively. The influence of an initial stress on the vibration of conducting medium amidst gravitational field forces was analysed by El-Naggar et al.^12^ and initially stressed multilayered piezoelectric composite bodies were examined in Qian et al.,^13^ while the propagation of surface elastic waves under thermal relaxation and voids presence was studied by Abo-Dahab et al.^14^ In addition, several investigations of the wave propagation in bodies and elastic media comprise the presence of viscous damping by Leissa and Qatu^15^ and the presence of material and structural discontinuities.^16,17^ Furthermore, the propagation and dispersion of waves in multilayered and composite structures were equally examined in the literature.^18–19^ Such structures include sandwich plates, layered laminates, composite rods, photovoltaic panels and beams to mention a few. For example, the harmonic wave assessment in an elastic sandwich plate was examined by Lee and Chang,^20^ and the dispersion of waves in an inhomogeneous elastic three-layered plate was considered Naumenko and Eremeyev.^21^ In addition, more relevant studies with regard to the layered media include the wave propagation in layered photovoltaic panels and laminated glass,^22^ buckling and bending analysis of vibrating composite and sandwich beams by Sayyad and Ghugal,^23^ the determination of lowest motion modes of elastic beams with alternating components by Sahin et al.,^24^ a layer-wise finite element analysis for composite plates by Belarbi et al.^25^ and for sandwich five-layered composites by Shishehsaz et al.^26^ More, the influence of certain forces on the propagation of waves in multilayered media was investigated including the propagation of waves in an inhomogeneous magneto-electro-hollow cylinder and on elastic plates by Zhu and Shi^27^ and Jiangong et al.,^28^ respectively, and the determination of an analytic solution to a Love wave problem in a double-layered media underlying an inhomogeneous half-space layer by Mandi et al.,^29^ to mention a few; see also previous studies^30–35^ and the references therein for more notable related works.

However, we explore in this article the influence of the thermal stress, magnetic field and rotational effects on the propagation of surface waves in an inhomogeneous symmetric five-layered plate. The plate which is presumed to be of isotropic alternating materials is prescribed with perfect interfaces and traction-free outer faces. Similarly, the expected related displacements and stresses will be determined in each layer of the plate in relation to both the symmetric and antisymmetric cases. Also, the determination of the dispersion relations and their analysis will be carried out, in addition to the determination of the respective cut-off frequencies. Moreover, this article goes as follows: in section “Governing equations,” the governing equations of motion in the presence of external forces and rotation are supplied. Section “Formulation of the problem” gives the formulation of the aiming problem. Section “The exact solution” presents the exact solutions to the problem, and section “The Dispersion relation” determined and analysed the dispersion relation, while section “Lowest cut-off frequency” determined the cut-off frequencies. The numerical results are given in section “Numerical results and interpretation,” with the conclusion in the final section.

## Governing equations

We consider an isotropic homogeneous elastic medium in the presence of magnetic fields, thermal stress and rotation. The governing equations then take the following forms:^7–12^

1. The strain–displacement relation

(1) $$\varepsilon_{\mathrm{ij}}=\frac{1}{2}(u_{j,i}+u_{i,j})$$

2. The stress–strain relation given by

(2) $$\sigma_{\mathrm{ij}}=\lambda \varepsilon_{\mathrm{kk}}\delta_{\mathrm{ij}}+2\mu \varepsilon_{\mathrm{ij}}$$

3. The equation of motion with magnetic force ${\vec{F}}_{i}$ and thermal stress ${\vec{G}}_{i}$

(3) $$\sigma_{\mathrm{ij},j}+{\vec{F}}_{i}+{\vec{G}}_{i}=\rho {\overset{\cdot \cdot }{u}}_{i}$$

where i,j=1,2,3 , ρ is the density, λ and μ are elastic constants, and $\delta_{\mathrm{ij}}$ is the Kronecker delta. Furthermore, the acceleration on the right-hand side of equation (3) in a rotating frame of reference with angular velocity Ω takes the following form:

4. The rotational acceleration

(4) $${\overset{\cdot \cdot }{u}}_{i}\to {\overset{\cdot \cdot }{u}}_{i}+\vec{\Omega }\times (\vec{\Omega }\times \vec{u})+2\vec{\Omega }\times {\overset{\cdot }{\vec{u}}}_{i}$$

where $\vec{\Omega }\times (\vec{\Omega }\times \vec{u})$ and $2\vec{\Omega }\times {\overset{\cdot }{\vec{u}}}_{i}$ are the centripetal and Coriolis accelerations, respectively.^35^

Furthermore, the linearized Maxwell equations for the electromagnetic field in a conducting medium take the forms

(5) $$\begin{array}{c} \mathrm{Div}\vec{H}=0,\;\mathrm{Div}\vec{E}=0\\ \mathrm{Curl}\vec{H}=\vec{J}\times \epsilon_{0}\overset{\cdot }{\vec{E}},\;\mathrm{Curl}\vec{E}=-\mu_{0}\overset{\cdot }{\vec{H}}\\ \vec{E}=\mu_{0}\left(\overset{\cdot }{\vec{u}}\times \vec{H}\right),\;\vec{h}=\mathrm{Curl}(\vec{u}\times \vec{H}) \end{array}$$

of which the magnetic field force takes the following form^34^

(6) $${\vec{F}}_{i}=\mu_{0}H_{0}^{2}\left(u_{j,\mathrm{ji}}-\epsilon_{0}\mu_{0}{\overset{\cdot \cdot }{u}}_{i}\right)$$

where $\epsilon_{0}$ is the electric field, $\mu_{0}$ is the magnetic permeability and $\vec{H}=H_{0}+\vec{h},\;\vec{h}$ is the induced magnetic field.

In addition, the thermal stress which was recently considered by Selim^7^ and Wang et al.^8^ takes the form

(7) $${\vec{G}}_{i}=-\left(\frac{\alpha \mathrm{Eh}}{1-\nu }\right)Tu_{,\mathrm{ii}}$$

where ν is Poisson’s ratio, E is Young’s Modulus, α is the thermal expansion coefficient and T is the temperature variation in the medium. Now, since Poisson’s ratio ν and Young’s Modulus E are given in terms of elastic constants as follows^15^

(8) $$\begin{array}{c} \nu \;=\frac{\lambda }{2(\lambda +\mu )}\\ E\;=\frac{\mu (3\lambda +2\mu )}{\lambda +\mu } \end{array}$$

equation (7) becomes

(9) $${\vec{G}}_{i}=-2\alpha h\left(\frac{3\lambda \mu +2\mu^{2}}{\lambda +2\mu }\right)Tu_{,\mathrm{ii}}$$

## Formulation of the problem

**Figure 1.**
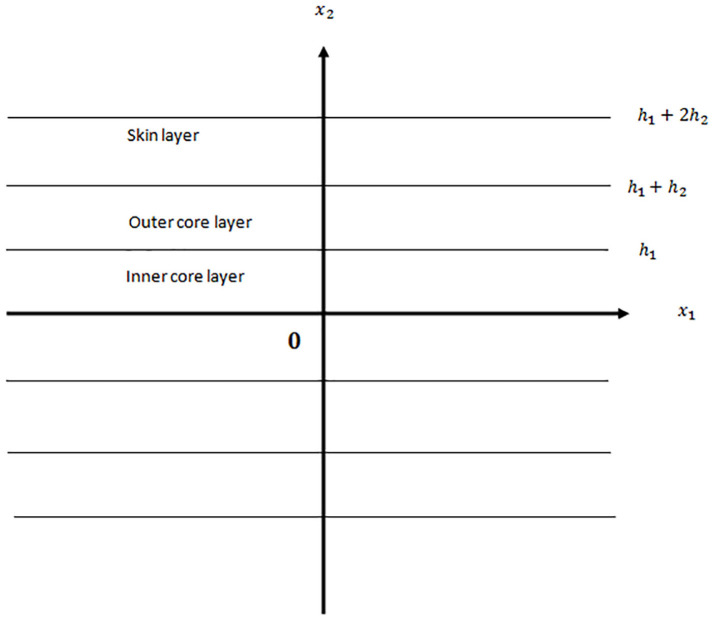
A symmetric magneto-rotator-five-layered elastic plate.

We consider in this section a magneto-rotator-five-layered plate made of isotropic inhomogeneous alternating layers as shown in Figure 1. The plate consists of the inner core layer of thickness of $2h_{1}$ , the outer core layer of thickness of $\left(h_{1}+h_{2}\right)$ and the skin layer of thickness of $\left(h_{1}+2h_{2}\right)$ . In addition, the plate is considered to be symmetrical about $x_{2}=0$ , and both the inner core layer and the skin layers are of the same material properties.

Consider the Cartesian coordinates system $\left(x_{1},x_{2},x_{3}\right)$ , then the displacements $\left(u_{1},u_{2},u_{3}\right)$ in $\left(x_{1},x_{2}\right)$ plane where t (time variable) are considered to be

(10) $$\begin{array}{c} u_{1}(x_{1},x_{2},t)\;=0\\ u_{2}(x_{1},x_{2},t)\;=0\\ u_{3}(x_{1},x_{2},t)\;=u \end{array}$$

The anti-plane shear motion^21^ in the above symmetric plate under consideration is governed by the following equations of motions from equations (1)–(10)

(11) $$\frac{\partial \sigma_{13}^{p}}{\partial x_{1}}+\frac{\partial \sigma_{23}^{p}}{\partial x_{2}}+N_{p}\left(\frac{\partial^{2}u^{p}}{\partial x_{1}^{2}}+\frac{\partial^{2}u^{p}}{\partial x_{2}^{2}}\right)=\rho_{p}\left(\frac{\partial^{2}u^{p}}{\partial t^{2}}-\Omega^{2}u^{p}\right)+\epsilon_{0}\mu_{0}^{2}H_{0}^{2}\frac{\partial^{2}u^{p}}{\partial t^{2}},\;\;p=i,o,s$$

for p=i,o,s stand for the inner core, outer core and skin layers, respectively, with $\rho_{i}=\rho_{s}$ , being alternating layers, and $\vec{\Omega }=\Omega (0,0,1),$ that is, the medium is assumed to rotate along $x_{3}$ axis; and $N_{q}(T)$ from equation (9) is

(12) $$N_{p}=-2\alpha_{p}h_{q}\mu_{p}\left(\frac{3\lambda_{p}+2\mu_{p}}{\lambda_{p}+2\mu_{p}}\right)T$$

p=1,2 . The shear stresses $\sigma_{j3}^{p},$ in equation (11), are obtained from equations (2) and (10) to be

(13) $$\sigma_{j3}^{p}=\mu_{p}\frac{\partial u^{p}}{\partial x_{j}},\;\;\;j=1,2,\;\;\;\;p=i,o,s$$

where $\mu_{i}=\mu_{s}$ .

Furthermore, we also define the following interfacial conditions of displacements and stresses

(14) $$\begin{array}{c} \;(I)\;u^{i}\left(x_{1},x_{2},t\right)=u^{o}\left(x_{1},x_{2},t\right),\;\mathrm{at}\;x_{2}\;=\pm h_{1}\\ (\mathrm{II})\;\sigma_{23}^{i}(x_{1},x_{2},t)=\sigma_{23}^{o}(x_{1},x_{2},t),\;\mathrm{at}\;x_{2}\;=\pm h_{1} \end{array}$$

(15) $$\begin{array}{c} \left(\mathrm{III})\;u^{o}\left(x_{1},x_{2},t\right)\;=u^{s}\left(x_{1},x_{2},t\right),\;\mathrm{at}\;x_{2}\;=\pm (h_{1}+h_{2}\right)\\ \left(\mathrm{IV})\;\sigma_{23}^{o}(x_{1},x_{2},t)=\sigma_{23}^{s}(x_{1},x_{2},t),\;\mathrm{at}\;x_{2}\;=\pm (h_{1}+h_{2}\right) \end{array}$$

and the following traction-free conditions

(16) $$\left(V)\;\sigma_{23}^{s}(x_{1},x_{2},t)=0,\;\mathrm{at}\;x_{2}=\pm (h_{1}+2h_{2}\right)$$

However, we determine in the subsequent section the related displacements and stresses in the respective layered of the plate by utilizing the prescribed interfacial and traction-free boundary conditions. Similarly, we will set modalities for determining the aimed dispersion relations for onward analysis.

## The exact solution

This section determines the exact solution of the formulated problem analytically using the normal mode method. By this, respective displacements and shear stresses will be determined in each layer of the symmetric plate under consideration.

Thus, equation (11) through equations (12) and (13) becomes the following

(17) $$\frac{\partial^{2}u^{p}}{\partial x_{1}^{2}}+\frac{\partial^{2}u^{p}}{\partial x_{2}^{2}}={\left(\frac{1}{S_{p}c_{p}}\right)}^{2}\left(\frac{\partial^{2}u^{p}}{\partial t^{2}}-\Omega^{2}u^{p}\right)+{\left(\frac{1}{S_{p}d_{p}}\right)}^{2}\frac{\partial^{2}u^{p}}{\partial t^{2}},\;\;\;\;\;p=i,o,s$$

where $c_{p}$ is the transverse speed, and

(18) $$\begin{array}{c} c_{p}\;=\sqrt{\frac{\mu_{p}}{\rho_{p}}}\\ d_{p}\;=\frac{1}{\mu_{0}H_{0}}\sqrt{\frac{\mu_{p}}{\epsilon_{0}}}\\ M_{T}\;=-2\alpha_{p}h_{p}\left(\frac{3\lambda_{p}+2\mu_{p}}{\lambda_{p}+2\mu_{p}}\right)T\\ S_{p}\;=\sqrt{M_{p}+1} \end{array}$$

Therefore, to determine the surface wave propagation in the plate, equation (17) via the normal mode method admits the following solution form

(19) $$u^{p}(x_{1},x_{2},t)=v^{p}(x_{2})e^{\mathrm{ik}(x_{1}-\mathrm{ct})},\;\;\;\;p=i,o,s$$

where k and c are the wave number and phase speed velocity, respectively; and $i=\sqrt{-1}$ . Consequently, equation (17) via (18) takes the following form

(20) $$\frac{d^{2}v^{p}}{dx_{2}}+k^{2}\left(R_{p}-1\right)v^{p}=0,\;\;\;\;\;p=i,o,s$$

where

(21) $$R_{p}={\left(\frac{c}{S_{p}c_{p}}\right)}^{2}+{\left(\frac{c}{S_{p}d_{p}}\right)}^{2}+{\left(\frac{\Omega }{S_{p}c_{p}k}\right)}^{2},\;\;\;\;\;p=i,o,s$$

Thus, we obtain the respective solutions in the symmetric five-layered plate as follows

(22) $$\begin{array}{c} \;v^{i}(x_{2})=A_{1}\cos\left(kx_{2}\sqrt{R_{i}-1}\right)+B_{1}\sin\left(kx_{2}\sqrt{R_{i}-1}\right),\;\;\;\;0\leq x_{2}\leq h_{1}\\ v^{o}(x_{2})=A_{2}\cos\left(kx_{2}\sqrt{R_{o}-1}\right)+B_{2}\sin\left(kx_{2}\sqrt{R_{o}-1}\right),\;\;\;\;h_{1}\leq x_{2}\leq (h_{1}+h_{2})\\ v^{s}(x_{2})=A_{3}\cos\left(kx_{2}\sqrt{R_{s}-1}\right)+B_{3}\sin\left(kx_{2}\sqrt{R_{s}-1}\right),\;\;\;\;(h_{1}+h_{2})\leq x_{2}\leq (h_{1}+2h_{2}) \end{array}$$

where $R_{i}=R_{s}$ , and $A_{n},B_{n}$ are unknowns for each layer n=1,2,3 to be computed from the prescribed continuity and boundary conditions. Note that since the plate is considered to be symmetric, we only considered the part of $x_{2}\geq 0$ ; one can equally consider the part of $x_{2}\leq 0$ which ultimately yields the same results.

### Symmetric solution

For the symmetric solution for $x_{2}\geq 0$ , we obtain the solutions from equation (22) corresponding to the inner core layer, the outer core layer, and the skin layer as follows

(23) $$\begin{array}{c} v^{i}(x_{2})=A_{1}\cos\left(kx_{2}\sqrt{R_{i}-1}\right),\;\;\;\;\;\;\;\;\;\;\;\;\;\;\;\;\;\;\;\;\;\;\;\;\;\;\;\;\;\;\;\;\;\;\;\;\;\;0\leq x_{2}\leq h_{1}\\ v^{o}(x_{2})=A_{2}\cos\left(kx_{2}\sqrt{R_{o}-1}\right)+B_{2}\sin\left(kx_{2}\sqrt{R_{o}-1}\right),\;\;\;\;h_{1}\leq x_{2}\leq (h_{1}+h_{2})\\ v^{o}(x_{2})=A_{2}\cos\left(kx_{2}\sqrt{R_{o}-1}\right)+B_{2}\sin\left(kx_{2}\sqrt{R_{o}-1}\right),\;\;\;\;h_{1}\leq x_{2}\leq (h_{1}+h_{2}) \end{array}$$

However, we determine below the complete exact displacements and stresses (in the absence of the exponential factor $e^{\mathrm{ik}(x_{1}-\mathrm{ct})}$ ) in the inner core layer, the outer core layer and the skin layer from the continuity and boundary conditions given in equations (14)–(16) and the solutions in equation (23) as follows

(24) $$\begin{array}{c} \;v^{i}=\;\cos\left(k\xi_{2}Q_{i}\right)\\ \sigma_{13}^{i}=\;i\mu_{i}k\cos\left(k\xi_{2}Q_{i}\right)\\ \sigma_{23}^{i}=\;-\mu_{i}kQ_{i}\sin\left(k\xi_{2}Q_{i}\right) \end{array}$$

(25) $$\begin{array}{c} \;v^{o}=\;\cos\left(h_{1}kQ_{i}\right)\cos\left(k\left(\zeta_{2}-h_{1}\right)Q_{o}\right)-\frac{\mu_{i}Q_{i}}{\mu_{o}Q_{o}}\sin\left(h_{1}kQ_{i}\right)\sin\left(k\left(\zeta_{2}-h_{1}\right)Q_{o}\right)\\ \sigma_{13}^{o}=\;i\mu_{o}k\left(\cos\left(h_{1}kQ_{i}\right)\cos\left(k\left(\zeta_{2}-h_{1}\right)Q_{o}\right)-\frac{\mu_{i}Q_{i}}{\mu_{o}Q_{o}}\sin\left(h_{1}kQ_{i}\right)\sin\left(k\left(\zeta_{2}-h_{1}\right)Q_{o}\right)\right)\\ \sigma_{23}^{o}=\;-\mu_{o}kQ_{o}\left(\cos\left(h_{1}kQ_{i}\right)\sin\left(k\left(\zeta_{2}-h_{1}\right)Q_{o}\right)+\frac{\mu_{i}Q_{i}}{\mu_{o}Q_{o}}\sin\left(h_{1}kQ_{i}\right)\cos\left(k\left(\zeta_{2}-h_{1}\right)Q_{o}\right)\right) \end{array}$$

and

(26) $$\begin{array}{c} \;v^{s}=\;\Theta \sec\left(h_{2}kQ_{s}\right)\cos\left(k\left(\eta_{2}-h_{1}-2h_{2}\right)Q_{s}\right)\\ \sigma_{13}^{s}=\;i\mu_{s}k\Theta \sec\left(h_{2}kQ_{s}\right)\cos\left(k\left(\eta_{2}-h_{1}-2h_{2}\right)Q_{s}\right)\\ \sigma_{23}^{s}=-\mu_{s}kQ_{s}\Theta \sec\left(h_{2}kQ_{s}\right)\sin\left(k\left(\eta_{2}-h_{1}-2h_{2}\right)Q_{s}\right) \end{array}$$

where

(27) $$\Theta =\cos\left(h_{1}kQ_{i}\right)\cos\left(h_{2}kQ_{o}\right)-\frac{\mu_{i}Q_{i}}{\mu_{o}Q_{o}}\sin\left(h_{1}kQ_{i}\right)\sin\left(h_{2}kQ_{o}\right)$$

and within the ranges below

(28) $$\begin{array}{c} \;0\leq \xi_{2}\leq h_{1}\\ \;h_{1}\leq \zeta_{2}\leq (h_{1}+h_{2})\\ \left(h_{1}+h_{2})\leq \eta_{2}\leq (h_{1}+2h_{2}\right) \end{array}$$

where

(29) $$Q_{i}=\sqrt{R_{T}^{i}-1},\;\;Q_{o}=\sqrt{R_{T}^{o}-1},\;\;Q_{s}=\sqrt{R_{T}^{s}-1}$$

### Antisymmetric solution

The antisymmetric solution for $x_{2}\geq 0$ from equation (22) reduces to the following form

(30) $$\begin{array}{c} v_{i}(x_{2})=B_{1}\sin\left(kx_{2}\sqrt{R_{i}-1}\right),\;\;\;\;\;\;\;\;\;\;\;\;\;\;\;\;\;\;\;\;\;\;\;\;\;\;\;\;\;\;\;\;\;\;\;\;\;\;0\leq x_{2}\leq h_{1}\\ v_{o}(x_{2})=A_{2}\cos\left(kx_{2}\sqrt{R_{o}-1}\right)+B_{2}\sin\left(kx_{2}\sqrt{R_{o}-1}\right),\;\;\;\;h_{1}\leq x_{2}\leq (h_{1}+h_{2})\\ v_{s}(x_{2})=A_{3}\cos\left(kx_{2}\sqrt{R_{s}-1}\right)+B_{3}\sin\left(kx_{2}\sqrt{R_{s}-1}\right),\;\;\;\;(h_{1}+h_{2})\leq x_{2}\leq (h_{1}+2h_{2}) \end{array}$$

Also, in the same passion, we determine exact displacements and stresses in the respective layers as follows

(31) $$\begin{array}{c} \;v^{i}=\sin\left(k\xi_{2}Q_{i}\right)\\ \sigma_{13}^{i}=i\mu_{i}k\sin\left(k\xi_{2}Q_{i}\right)\\ \sigma_{23}^{i}=\mu_{i}kQ_{i}\cos\left(k\xi_{2}Q_{i}\right) \end{array}$$

(32) $$\begin{array}{c} \;v^{o}=\sin\left(h_{1}kQ_{i}\right)\cos\left(k\left(\zeta_{2}-h_{1}\right)Q_{o}\right)+\frac{\mu_{i}Q_{i}}{\mu_{o}Q_{o}}\cos\left(h_{1}kQ_{i}\right)\sin\left(k\left(\zeta_{2}-h_{1}\right)Q_{o}\right)\\ \sigma_{13}^{o}=i\mu_{o}k\left(\sin\left(h_{1}kQ_{i}\right)\cos\left(k\left(\zeta_{2}-h_{1}\right)Q_{o}\right)+\frac{\mu_{i}Q_{i}}{\mu_{o}Q_{o}}\cos\left(h_{1}kQ_{i}\right)\sin\left(k\left(\zeta_{2}-h_{1}\right)Q_{o}\right)\right)\\ \sigma_{23}^{o}=-\mu_{o}kQ_{o}\left(\sin\left(h_{1}kQ_{i}\right)\sin\left(k\left(\zeta_{2}-h_{1}\right)Q_{o}\right)-\frac{\mu_{i}Q_{i}}{\mu_{o}Q_{o}}\cos\left(h_{1}kQ_{i}\right)\cos\left(k\left(\zeta_{2}-h_{1}\right)Q_{o}\right)\right) \end{array}$$

and

(33) $$\begin{array}{c} \;v^{s}=\Phi \sec\left(h_{2}kQ_{s}\right)\cos\left(k\left(\eta_{2}-h_{1}-2h_{2}\right)Q_{s}\right)\\ \sigma_{13}^{s}=i\mu_{s}k\Phi \sec\left(h_{2}kQ_{s}\right)\cos\left(k\left(\eta_{2}-h_{1}-2h_{2}\right)Q_{s}\right)\\ \sigma_{23}^{s}=-\mu_{s}kQ_{s}\Phi \sec\left(h_{2}kQ_{s}\right)\sin\left(k\left(\eta_{2}-h_{1}-2h_{2}\right)Q_{s}\right) \end{array}$$

with

(34) $$\begin{array}{c} \Phi =\sin\left(h_{1}kQ_{i}\right)\cos\left(h_{2}kQ_{o}\right)+\frac{\mu_{i}Q_{i}}{\mu_{o}Q_{o}}\cos\left(h_{1}kQ_{i}\right)\sin\left(h_{2}kQ_{o}\right) \end{array}$$

where $\xi_{2},\;\zeta_{2}$ and $\eta_{2}$ are defined over the ranges given in equation (28), and $Q_{p},\;(p=i,o,s)$ is also given in equation (29).

## The dispersion relation

This section determines the generalized dispersion relation for the formulated problem corresponding to both the symmetric and antisymmetric solutions modes presented earlier.

### Symmetric dispersion relation

Here, the dispersion relation of the formulated problem associated with the symmetric solution modes is presented.

In doing so, the symmetric modes via the use of the continuity and boundary conditions prescribed in equations (14)–(16) and the solutions obtained equation (22) yield a 5 × 5 dispersion matrix of which

(35) $$\left|\begin{array}{ccccc} a_{11} & a_{12} & a_{13} & 0 & 0\\ a_{21} & a_{22} & a_{23} & 0 & 0\\ 0 & a_{32} & a_{33} & a_{34} & a_{35}\\ 0 & a_{42} & a_{43} & a_{44} & a_{45}\\ 0 & 0 & 0 & a_{54} & a_{55} \end{array}\right|\;=0$$

where

$$\begin{array}{c} a_{11}=\cos\left(kh_{1}Q_{i}\right),\;a_{12}=-\cos\left(kh_{1}Q_{o}\right)\\ a_{13}=-\sin\left(kh_{1}Q_{o}\right),\;a_{21}=\frac{Q_{i}\mu_{i}}{Q_{o}\mu_{o}}\sin\left(kh_{1}Q_{i}\right)\\ a_{22}=\cos\left(kh_{1}Q_{o}\right),\;a_{23}=-\sin\left(kh_{1}Q_{o}\right)\\ a_{32}=\cos\left(k\left(h_{1}+h_{2}\right)Q_{o}\right),\;a_{33}=\sin\left(k\left(h_{1}+h_{2}\right)Q_{o}\right)\\ a_{34}=-\cos\left(k\left(h_{1}+h_{2}\right)Q_{s}\right),\;a_{35}=-\sin\left(k\left(h_{1}+h_{2}\right)Q_{s}\right)\\ a_{42}=\sin\left(k\left(h_{1}+h_{2}\right)Q_{o}\right),\;a_{43}=-\cos\left(k\left(h_{1}+h_{2}\right)Q_{o}\right)\\ a_{44}=-\frac{Q_{s}\mu_{s}}{Q_{o}\mu_{o}}\sin\left(k\left(h_{1}+h_{2}\right)Q_{s}\right),\;a_{45}=\frac{Q_{s}\mu_{s}}{Q_{o}\mu_{o}}\cos\left(k\left(h_{1}+h_{2}\right)Q_{s}\right)\\ a_{54}=\sin\left(k\left(h_{1}+2h_{2}\right)Q_{s}\right),\;a_{55}=-\cos\left(k\left(h_{1}+2h_{2}\right)Q_{s}\right) \end{array}$$

with $Q_{i}=Q_{s}$ .

Thus, the dispersion relation corresponding to the symmetric modes is obtained (equation (35)) as follows

(36) $$\begin{array}{c} \mu_{i}Q_{i}\mu_{o}Q_{o}\left(\tan\left(h_{1}kQ_{i}\right)\cos\left(h_{2}kQ_{o}\right)-\tan\left(h_{2}kQ_{i}\right)\sin\left(\left(2h_{1}+h_{2}\right)kQ_{o}\right)\right)\\ +\mu_{o}^{2}Q_{o}^{2}\cos\left(\left(2h_{1}+h_{2}\right)kQ_{o}\right)-\mu_{i}^{2}Q_{i}^{2}\tan\left(h_{1}kQ_{i}\right)\tan\left(h_{2}kQ_{i}\right)\sin\left(h_{2}kQ_{o}\right)=0 \end{array}$$

#### Special case

However, a very special case of the obtained dispersion relation for symmetric modes given above will be when all these external quantities are absent, that it, when the rotation Ω→0 , magnetic field $H_{0}\to 0$ and the temperature variation T→0 , which reduces to

(37) $$\begin{array}{c} \mu_{i}W_{i}\mu_{o}W_{o}\left(\tan\left(h_{1}kW_{i}\right)\cos\left(h_{2}kW_{o}\right)-\tan\left(h_{2}kW_{i}\right)\sin\left(\left(2h_{1}+h_{2}\right)kW_{o}\right)\right)\\ +\mu_{o}^{2}W_{o}^{2}\cos\left(\left(2h_{1}+h_{2}\right)kW_{o}\right)-\mu_{i}^{2}W_{i}^{2}\tan\left(h_{1}kW_{i}\right)\tan\left(h_{2}kW_{i}\right)\sin\left(h_{2}kW_{o}\right)=0 \end{array}$$

where

(38) $$W_{i}=\sqrt{\frac{c^{2}}{c_{i}^{2}}-1},\;\;\;\;\;W_{o}=\sqrt{\frac{c^{2}}{c_{o}^{2}}-1}$$

### Antisymmetric dispersion relation

Proceeding as above, the dispersion relation for the antisymmetric modes gave a 5 × 5 dispersion matrix of which

(39) $$\left|\begin{array}{ccccc} b_{11} & b_{12} & b_{13} & 0 & 0\\ b_{21} & b_{22} & b_{23} & 0 & 0\\ 0 & b_{32} & b_{33} & b_{34} & b_{35}\\ 0 & b_{42} & b_{43} & b_{44} & b_{45}\\ 0 & 0 & 0 & b_{54} & b_{55} \end{array}\right|=0$$

with the following entries

$$\begin{array}{c} b_{11}=\sin\left(kh_{1}Q_{i}\right),\;b_{12}=-\cos\left(kh_{1}Q_{o}\right)\\ b_{13}=-\sin\left(kh_{1}Q_{o}\right),\;b_{21}=\frac{Q_{i}\mu_{i}}{Q_{o}\mu_{o}}\cos\left(kh_{1}Q_{i}\right)\\ b_{22}=\sin\left(kh_{1}Q_{o}\right),\;b_{23}=-\cos\left(kh_{1}Q_{o}\right)\\ b_{32}=\cos\left(k\left(h_{1}+h_{2}\right)Q_{o}\right),\;b_{33}=\sin\left(k\left(h_{1}+h_{2}\right)Q_{o}\right)\\ b_{34}=-\cos\left(k\left(h_{1}+h_{2}\right)Q_{s}\right),\;b_{35}=-\sin\left(k\left(h_{1}+h_{2}\right)Q_{s}\right)\\ b_{42}=\sin\left(k\left(h_{1}+h_{2}\right)Q_{o}\right),\;b_{43}=-\cos\left(k\left(h_{1}+h_{2}\right)Q_{o}\right)\\ b_{44}=-\frac{Q_{s}\mu_{s}}{Q_{o}\mu_{o}}\sin\left(k\left(h_{1}+h_{2}\right)Q_{s}\right),\;b_{45}=\frac{Q_{s}\mu_{s}}{Q_{o}\mu_{o}}\cos\left(k\left(h_{1}+h_{2}\right)Q_{s}\right)\\ b_{54}=\sin\left(k\left(h_{1}+2h_{2}\right)Q_{s}\right),\;b_{55}=-\cos\left(k\left(h_{1}+2h_{2}\right)Q_{s}\right) \end{array}$$

where $Q_{i}=Q_{s}.$

Accordingly, the antisymmetric dispersion relation is obtained from equation (39) as follows

(40) $$\begin{array}{c} \mu_{i}Q_{i}\mu_{o}Q_{o}\cos\left(\left(h_{1}+h_{2}\right)kQ_{i}\right)\cos\left(h_{2}kQ_{o}\right)-\mu_{i}^{2}Q_{i}^{2}\sin\left(h_{2}kQ_{i}\right)\cos\left(h_{1}kQ_{i}\right)\sin\left(h_{2}kQ_{o}\right)\\ -\mu_{o}^{2}Q_{o}^{2}\sin\left(h_{1}kQ_{i}\right)\cos\left(h_{2}kQ_{i}\right)\sin\left(h_{2}kQ_{o}\right)=0 \end{array}$$

#### Special case

Also, a special case of the obtained dispersion relation for antisymmetric modes given above will be in the absence of the rotation, magnetic field, and the temperature variation; that is, when Ω→0 , $H_{0}\to 0$ and T→0 to get

(41) $$\begin{array}{c} \mu_{i}W_{i}\mu_{o}W_{o}\cos\left(\left(h_{1}+h_{2}\right)kW_{i}\right)\cos\left(h_{2}kW_{o}\right)-\mu_{i}^{2}W_{i}^{2}\sin\left(h_{2}kW_{i}\right)\cos\left(h_{1}kW_{i}\right)\sin\left(h_{2}kW_{o}\right)\\ -\mu_{o}^{2}W_{o}^{2}\sin\left(h_{1}kW_{i}\right)\cos\left(h_{2}kW_{i}\right)\sin\left(h_{2}kW_{o}\right)=0 \end{array}$$

where $W_{i}$ and $W_{o}$ are given in equation (39).

## Lowest cut-off frequency

This section determines the cut-off frequencies associated with both the symmetric and antisymmetric dispersion relations obtained in the previous section. However, to determine the lowest cut-off frequency, we recall from equation (19) that ω=kc , such that

(42) $$c=\frac{\omega }{k}$$

where ω,k and c are the frequency, wave number and phase speed velocity, respectively.

Thus, with this relation defined in equation (42), $R_{p}$ from equation (21) then takes the following form

(43) $$R_{p}=\frac{1}{k^{2}}\left({\left(\frac{\omega }{S_{p}c_{p}}\right)}^{2}+{\left(\frac{\omega }{S_{p}d_{p}}\right)}^{2}+{\left(\frac{\Omega }{S_{p}c_{p}}\right)}^{2}\right),\;\;\;\;\;p=i,o,s$$

Also, from equation (29) via equation (43), we get

(44) $$Q_{p}=\frac{1}{k}\sqrt{Z_{p}^{2}-k^{2}}$$

where

(45) $$\begin{array}{cc} Z_{p}= & \sqrt{\omega^{2}V_{p}^{2}+{\left(\frac{\Omega }{S_{p}c_{p}}\right)}^{2}}\\ V_{p}= & \sqrt{{\left(\frac{1}{S_{p}c_{p}}\right)}^{2}+{\left(\frac{1}{S_{p}d_{p}}\right)}^{2}} \end{array}$$

for p=i,o,s.

Therefore, the *Cut-off frequency for the symmetric modes* is obtained from the symmetric dispersion relation given in equation (36) with the help of relations equations (42)–(45) and by setting k=0 as

(46) $$\begin{array}{c} \mu_{i}Z_{i}\mu_{o}Z_{o}\tan\left(h_{1}Z_{i}\right)\cos\left(h_{2}Z_{o}\right)-\mu_{i}Z_{i}\mu_{o}Z_{o}\tan\left(h_{2}Z_{i}\right)\sin\left(\left(2h_{1}+h_{2}\right)Z_{o}\right)\\ +\mu_{o}^{2}Z_{o}^{2}\cos\left(\left(2h_{1}+h_{2}\right)Z_{o}\right)-\mu_{i}^{2}Z_{i}^{2}\tan\left(h_{1}Z_{i}\right)\tan\left(h_{2}Z_{i}\right)\sin\left(h_{2}Z_{o}\right)=0 \end{array}$$

Also, for the antisymmetric modes, we get the corresponding *Cut-off frequency* from the antisymmetric dispersion relation given in equation (40) as follows

(47) $$\begin{array}{c} \mu_{i}Z_{i}\mu_{o}Z_{o}\cos\left(\left(h_{1}+h_{2}\right)Z_{i}\right)\cos\left(h_{2}Z_{o}\right)-\mu_{o}^{2}Z_{o}^{2}\sin\left(h_{1}Z_{i}\right)\cos\left(h_{2}Z_{i}\right)\sin\left(h_{2}Z_{o}\right)\\ -\mu_{i}^{2}Z_{i}^{2}\sin\left(h_{2}Z_{i}\right)\cos\left(h_{1}Z_{i}\right)\sin\left(h_{2}Z_{o}\right)=0 \end{array}$$

together with the predicted *single cut-off frequency* as

(48) $$\omega =\sqrt{\frac{c_{o}^{2}\mu_{i}S_{o}^{2}\left(c_{i}^{2}S_{i}^{2}\mu_{o}-h_{2}^{2}\Omega^{2}\mu_{i}\right)-h_{1}h_{2}\Omega^{2}c_{i}^{2}S_{i}^{2}\mu_{o}^{2}}{h_{2}c_{i}^{2}c_{o}^{2}S_{i}^{2}S_{o}^{2}\left(h_{2}\mu_{i}^{2}V_{i}^{2}+h_{1}\mu_{o}^{2}V_{o}^{2}\right)}}$$

It is worth noting here that the antisymmetric modes are known to possess global low-frequency regime whenever ω<<1; whereas the symmetric modes do not support that, see Prikazchikov et al.^21^ and Sergushova^36^ for the cases of three-layered laminate and rods. Also, due to the number of external excitations considered, the global low-frequency inequality for the antisymmetric modes cannot be explicitly determined, but numerically.

## Numerical results and interpretation

This section attempts to present the obtained results numerically by considering some physical data of interest together with a possible interpretation of the simulated results. In doing so, the derived dispersion relations for the symmetric and antisymmetric solution modes will be simulated to assess the effects of the rotation, magnetic field and temperature variation associated with the thermal stress. We start off by choosing the following thickness values of the inner core layer, outer core layer and skin layer as follows: $h_{1}=0.2\;m$ and $h_{2}=0.5\;m$ . Remember here that the outer core and skin layers are initially assumed to be of the same thickness. Furthermore, the five-layered plate is made up of alternating components or layers, thus we consider the inner core layer to be of copper material, the outer core layer of aluminium material and the skin layer of copper material. We also fix the wavenumber to be k=0.01 with the following values for c (considering the speed of light), $\epsilon_{0}$ (electric field) and $\mu_{0}$ (magnetic permeability) as used in Selim^7^ as follows

(49) $$c=2.998\times 10^{8}\;ms^{-1},\;\;\epsilon_{0}=8.85\times 10^{-12},\;\;\mu_{0}=4\pi \times 10^{-7}$$

Also, for the copper material, we obtain the following data^33,37^

(50) $$\begin{array}{c} \rho_{p}=8.954\times 10^{3}\;\mathrm{kg}\;m^{-3},\;\;\lambda_{p}=7.76\times 10^{10}N\;m^{-2},\;\;\mu_{p}=3.86\times 10^{10}N\;m^{-2},\\ \alpha_{p}=1.65\times 10^{-5}K^{-1} \end{array}$$

for p=i,s , and also for aluminium material with^34^

(51) $$\begin{array}{c} \rho_{p}=2.66\times 10^{3}\;\mathrm{kg}\;m^{-3},\;\;\lambda_{p}=5.65\times 10^{10}N\;m^{-2},\;\;\mu_{p}=2.46\times 10^{10}N\;m^{-2},\\ \;\;\alpha_{p}=2.31\times 10^{-5}K^{-1} \end{array}$$

for p=o. Below, we give some graphical illustrations and discussions. Figures 2–6 are obtained from the fundamental mode of the symmetric dispersion relation, while Figures 7–11 are for the corresponding antisymmetric mode.

**Figure 2.**
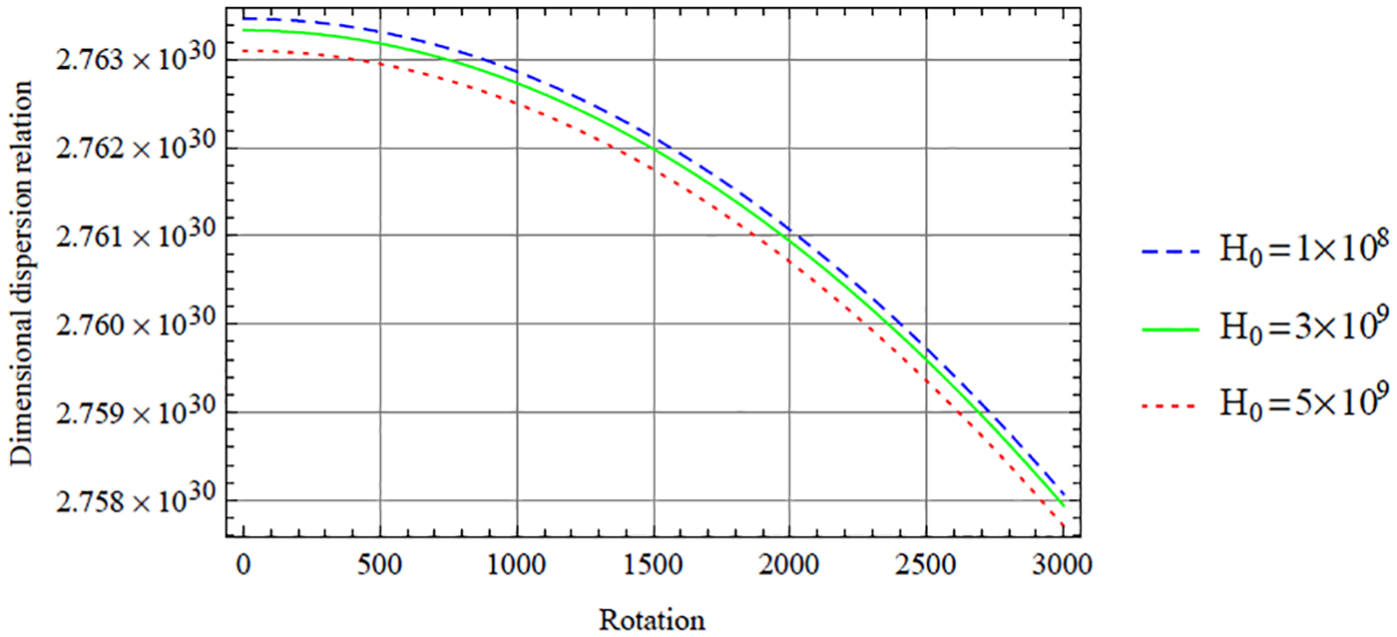
Variation of the symmetric dispersion relation given in equation (36) with respect to the rotation with variation in magnetic field.

**Figure 3.**
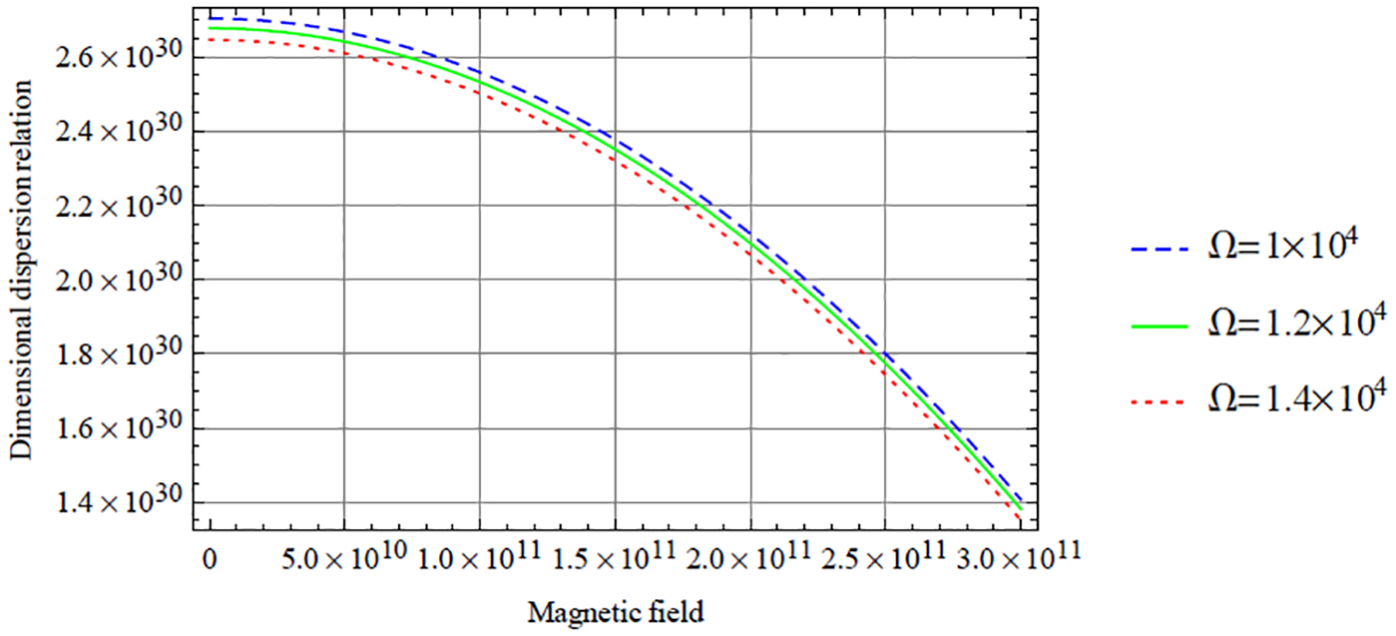
Variation of the symmetric dispersion relation given in equation (36) with respect to the magnetic field with variation in rotation.

**Figure 4.**
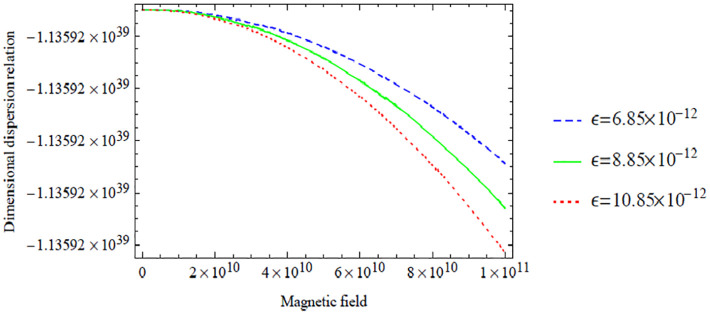
Variation of the symmetric dispersion relation given in equation (36) with respect to the magnetic field with variation in electric field.

**Figure 5.**
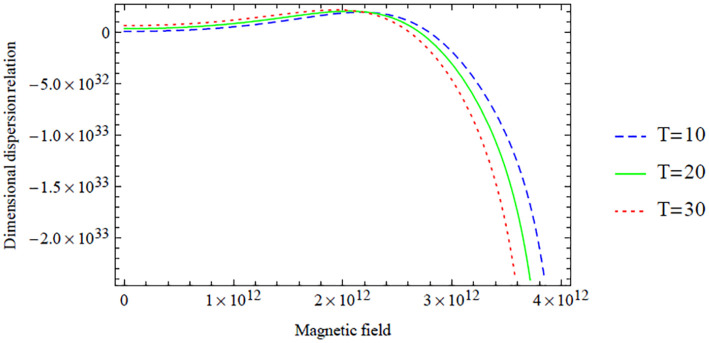
Variation of the symmetric dispersion relation given in equation (36) with respect to the magnetic field with temperature variation.

**Figure 6.**
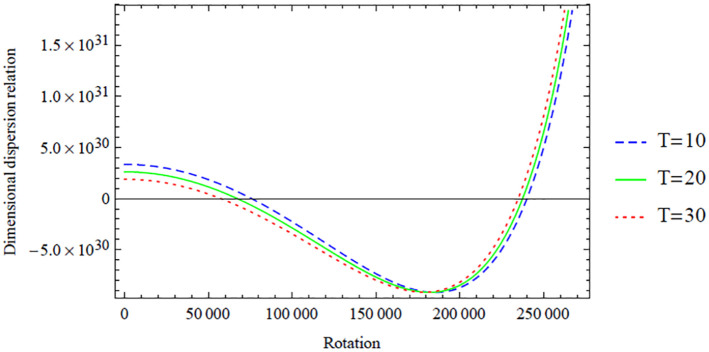
Variation of the symmetric dispersion relation given in equation (36) with respect to the rotation with temperature variation.

**Figure 7.**
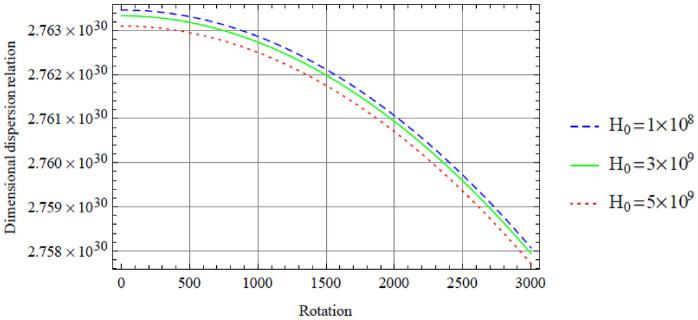
Variation of the antisymmetric dispersion relation given in equation (40) with respect to the rotation with variation in magnetic field.

**Figure 8.**
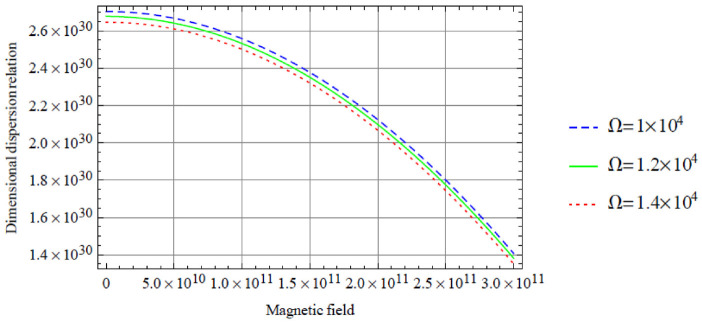
Variation of the antisymmetric dispersion relation given in equation (40) with respect to the magnetic field with variation in rotation.

**Figure 9.**
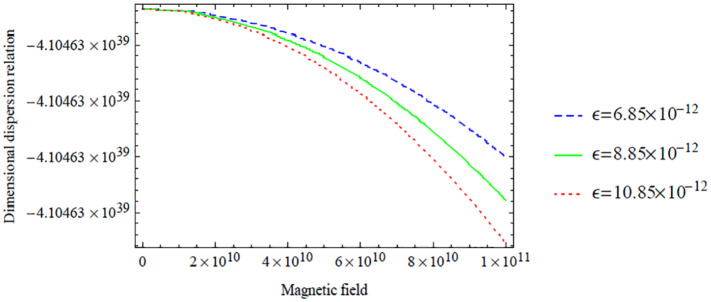
Variation of the antisymmetric dispersion relation given in equation (40) with respect to the magnetic field with variation in electric field.

**Figure 10.**
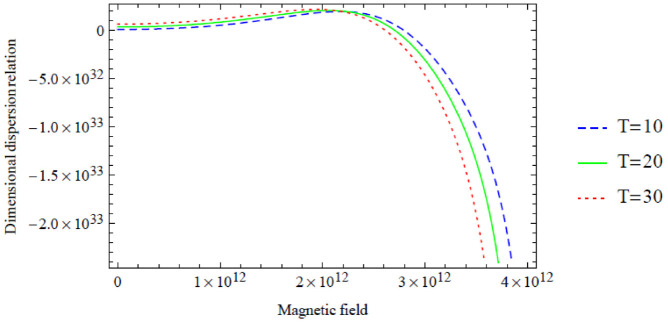
Variation of the antisymmetric dispersion relation given in equation (40) with respect to the magnetic field with temperature variation.

**Figure 11.**
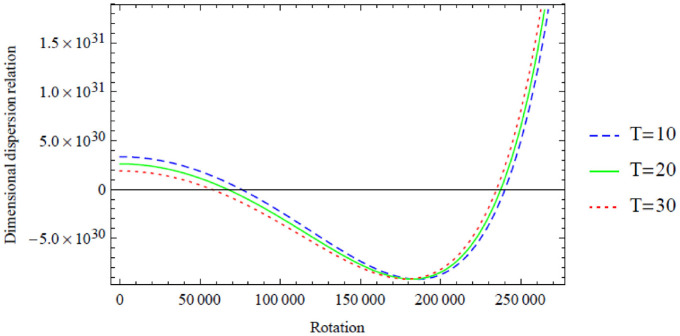
Variation of the antisymmetric dispersion relation given in equation (40) with respect to the rotation with temperature variation.

Figure 2 displays the variation of the dimensional symmetric dispersion relation given in equation (36) with respect to the rotation with variation in magnetic field, with temperature variation fixed at T=20 . It is noted that an increase in a magnetic field $H_{0}$ results in a decrease in dispersion curves with respect to the rotation. However, this decrease can be seen to be in a close range looking at the dimensional dispersion relation axis, and over a wide range for the rotation axis.

Figure 3 depicts the variation of the dimensional symmetric dispersion relation given in equation (36) with respect to the magnetic field with variation in rotation Ω , with temperature variation fixed at T=20 . In this case, a similar interpretation of Figure 2 can also be applied here. However, there is a significant decrease in the dimensional dispersion relation with respect to the magnetic field. It is important to also note that these curves are not visible when Ω is chosen below 1 × 10^4^, alongside a wide range of the magnetic field.

Figure 4 shows the variation of the dimensional symmetric dispersion relation given in equation (36) with respect to the magnetic field with variation in electric field ε , with temperature variation fixed at T=20 . Here, the dispersion relation curves are seen decreasing sharply with an increase in the electric field with respect to the magnetic field $H_{0}$ . Also, a significant decrease is noticed when the magnetic field is bigger than 2×10^10^.

Figure 5 showcases the variation of the dimensional symmetric dispersion relation given in equation (36) with respect to the magnetic field, with temperature variation at fixed rotation $\Omega =1\times 10^{7}$ . The dimensional dispersion relation curves are seen increasing almost linearly before a sudden significant decrease with an increase in temperature variation. It is further observed that even though the dispersion curves begin to increase with an increase in temperature, the sudden decrease tends to reverse the process of which high-temperature variation lags behind.

In Figure 6, the variation of the dimensional symmetric dispersion relation given in equation (36) with respect to the rotation with temperature variation, at fixed a magnetic field $H=1\times 10^{11}$ is showcased. The dimensional dispersion relation curves are seen decreasing continuously before a sudden significant increase with an increase in temperature variation. It is noticed here that even though the dispersion curves begin to decrease with an increase in temperature, the sudden increase reverses the process of which high-temperature variation begins to lead ahead.

However, a similar interpretation of the dimensional symmetric dispersion relation graphs shown in Figures 2–6 can also be drawn on the dimensional antisymmetric dispersion relation graphs shown in Figures 7–11. Nevertheless, it is worth noting here that the dispersion relation associated with the antisymmetric modes supports the long-wave low-frequency propagation, which leads to the possession of the global low-frequency regime; whereas the symmetric modes do not support that, see Prikazchikov et al.^21^ and Sergushova^36^ for more and some asymptotic approximations of the dispersion relation in the absence of external forces.

## Conclusion

In conclusion, this article explored the influence of the thermal stress on the propagation of surface waves in an inhomogeneous symmetric five-layered plate assumed to be in a rotating frame of reference in the presence of a magnetic field force. The layers of the plate are further presumed to be of alternating material constituents comprising densities and stiffnesses with suitable perfect interfacial conditions and traction-free boundaries on the outer faces. Furthermore, the respective displacements and stresses have been determined in each homogeneous layer. Besides, the resulting dispersion relations in both the symmetric and antisymmetric modes cases have been determined and analysed with emphasis on the variations on the magnetic field, rotation, electric field and temperature variation.

For the sake of the numerical simulation, a five-layered plate composed of alternating copper–aluminium layers is considered and analysed in regard to symmetric and antisymmetric fundamental mode cases of the dispersion relations when $x_{0}\geq 0$ . Indeed, from the obtained plots, the variations in thermal stress positively affect the wave propagation in the plate even for relatively smaller values of the temperature variation. Also, the same trend is observed in the case of the magnetic field, but for higher values. Finally, for the rotation, it was found that the angular velocity must reach at least 10^4^ before a significant change is observed, which in fact differs from the situations of half-plane reflection and single-layered plate propagation problems.
