# Corrigendum

**DOI:** 10.1177/00368504211017244

**Published:** 2021-05-11

Corrigendum to Effects of thermal stress, magnetic field and rotation on the dispersion of elastic waves in an inhomogeneous five-layered plate with alternating components. Science Progress 103(3): 1—22. DOI: 10.1177/0036850420940469

Figures 7 to11 were misprinted in the article. The correct figures should be read as follows:

**Figure 7.**
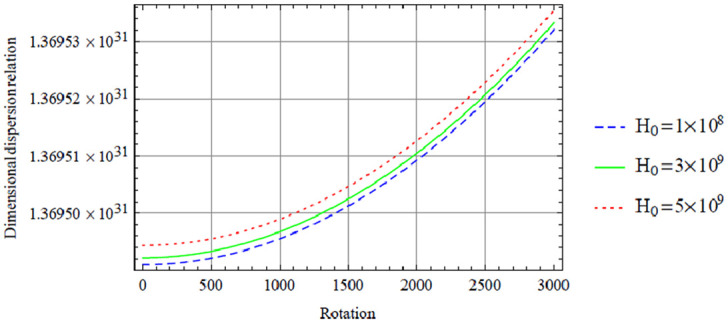
Variation of the antisymmetric dispersion relation given in equation (40) with respect to the rotation with variation in magnetic field.

**Figure 8.**
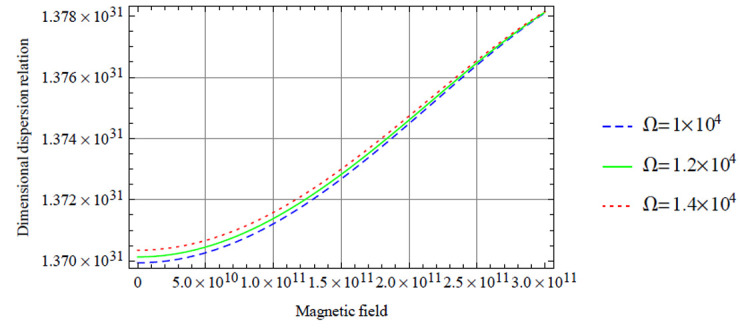
Variation of the antisymmetric dispersion relation given in equation (40) with respect to the magnetic field with variation in rotation.

**Figure 9.**
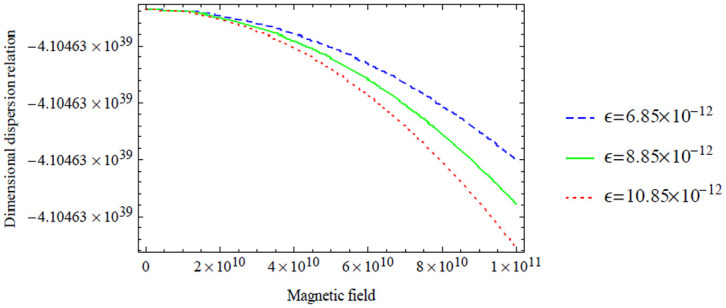
Variation of the antisymmetric dispersion relation given in equation (40) with respect to the magnetic field with variation in electric field.

**Figure 10.**
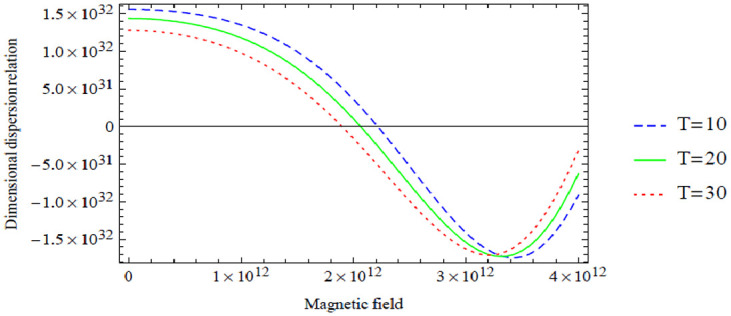
Variation of the antisymmetric dispersion relation given in equation (40) with respect to the magnetic field with temperature variation.

**Figure 11.**
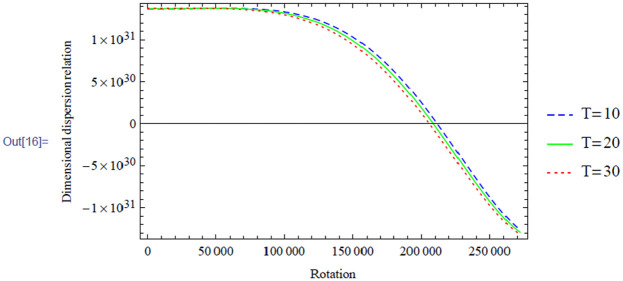
Variation of the antisymmetric dispersion relation given in equation (40) with respect to the rotation with temperature variation.

